# Fundamental Building Blocks for Scalable Photonic Programmable Processors

**DOI:** 10.1002/nap2.70100

**Published:** 2026-04-22

**Authors:** Cristina Gómez‐Hidalgo, Juan Fernández‐Vicente, Natalia Balbastre‐Benavent, María Manglano‐Bermejo, Marios Papadovasilakis, Ahmed Elsharabasy, Rocío Baños‐López, Jesús Benítez‐González, Ana Gutiérrez‐Campo, Tsjerk Hoekstra, Daniel Pérez‐López, Luis Torrijos‐Morán

**Affiliations:** ^1^ iPronics Programmable Photonics S.L. Valencia Spain; ^2^ Universitat Politecnica de Valencia Valencia Spain

**Keywords:** large‐scale silicon photonics, optical networking, optical switches, programmable photonic processors

## Abstract

Photonics technology is emerging as a key enabler, complementing electronics in next‐generation applications by offering greater bandwidth, lower latency and improved energy efficiency. Silicon photonic programmable processors bring these advantages to a solid‐state platform, providing compact form factors and low power consumption, although the scalability of densely integrated circuits is still under active investigation. In this article, we present a comprehensive study of the essential photonic components required for large‐scale integration of silicon photonic processors, covering both design and experimental demonstration. We analyse and optimise the fundamental building blocks—including splitters, phase shifters, and crossings—and experimentally validate their scalability through wafer‐scale characterisation. To finish, we illustrate the impact at the circuit level in a 4×4 optical switch loaded with high‐speed transmission and discuss the remaining challenges for large‐scale integration.

## Graphical abstract

Silicon photonic programmable processors are advanced through the optimisation and experimental validation of key building blocks, including splitters, phase shifters and crossings. In this work, their scalability is experimentally demonstrated at the wafer scale, and their circuit‐level impact is showcased in a high‐speed 4 × 4 optical switch, highlighting the pathway and remaining challenges towards large‐scale integration.

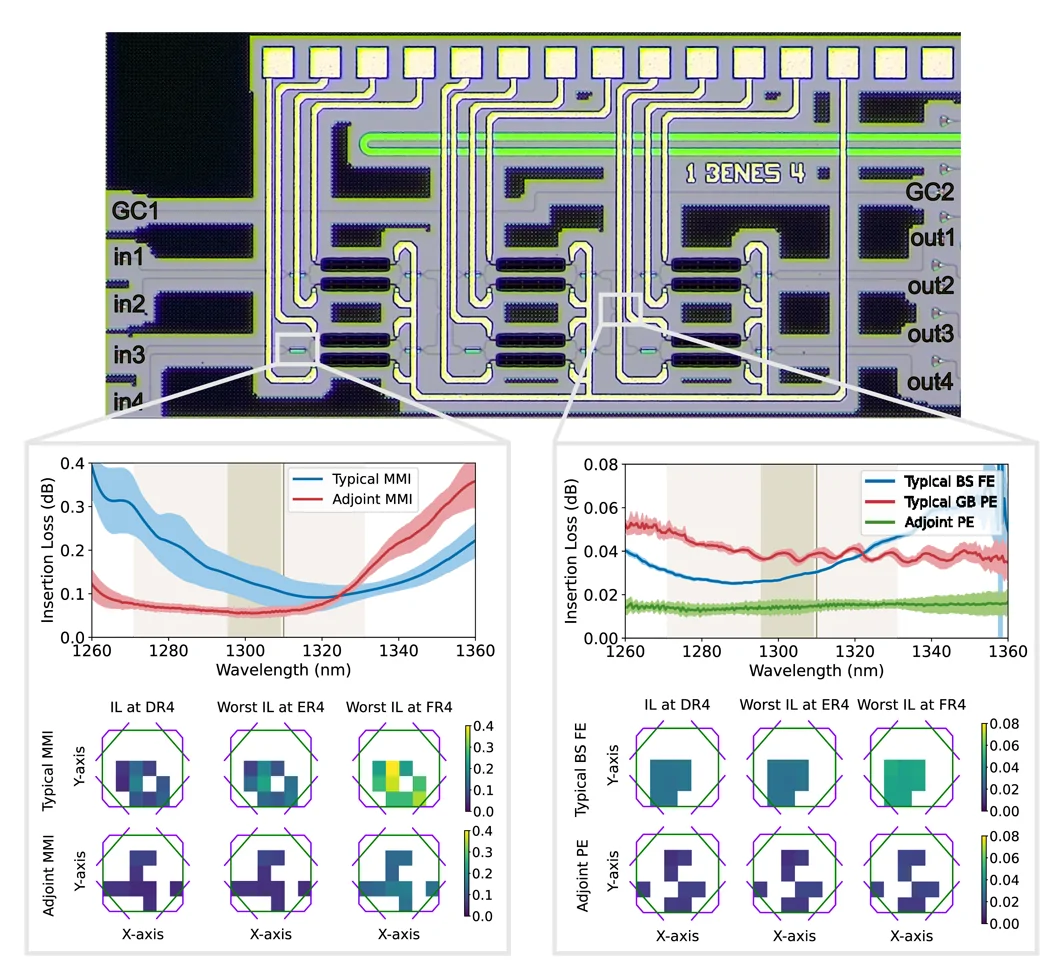

## 1 Introduction

Digital electronics have driven progress in computation and communications for decades, but as transistors approach atomic‐scale dimensions, challenges such as interconnect delay, leakage current and heat dissipation are increasingly constraining performance and energy efficiency [1]. At the same time, emerging applications such as 5G/6G networks [2], quantum processing [3] and artificial intelligence [4], demand higher bandwidth, lower latencies and scalability, pushing conventional CMOS technology to its fundamental limits.

In this context, programmable photonic processors have emerged as a powerful complementary approach to extend system performance beyond electronic limits. They exploit the intrinsic properties of light—ultra‐high bandwidth, low latency and parallelism via wavelength multiplexing—while integrating reconfigurability using interferometric meshes that can perform arbitrary linear transformations [5, 6]. This enables a wide range of applications within a single chip, including optical switching fabrics [7, 8], microwave photonic filters [9], beam‐forming networks [10, 11], matrix multiplication engines for AI acceleration [12], unitary quantum transformations [13, 14] and photonic neural networks [15, 16]. In particular, silicon photonic programmable processors combine these advantages with standard semiconductor process platforms that support large‐scale integration of active and passive components [17]. Although the scalability laws and fundamental limitations of programmable photonic processors have already been studied in [18], the overall performance of such large‐scale programmable systems is ultimately constrained by their fundamental building blocks that are massively repeated across the processor—power splitters, phase shifters, and crossings. Therefore, enhancing the performance and wafer‐scale reliability of these components is critical to realize large‐scale silicon photonic processors that meet the demanding requirements of next‐generation industrial applications.

In this work, we present a comprehensive study of the key building blocks that enable large‐scale silicon photonic processors, focusing on both the tuneable basic units and the passive structures interconnecting all components within the processor. We first review and simulate these components to identify the most promising candidates for dense integration. We then provide wafer‐scale experimental results to assess their reliability and demonstrate high performance. Finally, we report high‐speed transmission of 400 Gbps signals through a 4‐port Benes optical circuit switch.

## 2 Design of Building Blocks

In this section, we describe the key building blocks involved in a programmable photonic processor design: 50:50 splitters, phase shifters and crossings. Later, we benchmark the main design and optimisation strategies for each case, focusing on the system performance against the networking transceiver standards and the scalability on the standard 220‐nm silicon‐on‐insulator (SOI) platform. In the end, guidelines are provided for future development of the photonic components.

### 2.1 50:50 Splitters

The optical spectral response of a programmable photonic processor is conditioned by the insertion loss (IL) and the splitting ratio (SR) of its power splitters, as these directly influence the overall loss, crosstalk, and broadband operation. IL accounts for the fraction of optical power transmitted through the device, whereas SR establishes how this power is distributed between the output ports. A power splitter is considered broadband when both the IL and the SR exhibit limited wavelength dependence, ensuring consistent power transmission and distribution over a wide spectral range. In terms of scalability, the main concerns for power splitters are footprint and robustness. Footprint refers to the physical area required for integration, whereas robustness reflects tolerance to wafer‐scale fabrication variability.

In the literature, three traditional families of optical power splitters can be identified: directional couplers (DCs), which rely on evanescent coupling between adjacent waveguides [19], multimode interferometers (MMIs), which operate based on self‐imaging [20, 21], and adiabatic couplers (ACs), based on gradual adiabatic mode evolution [22]. In terms of performance, DCs typically exhibit broadband low‐loss operation but have a narrowband splitting ratio. MMIs show the opposite trend, with broadband splitting ratios but more bandwidth‐limited insertion loss. Regarding scalability, both DCs and MMIs can achieve compact footprints, although MMIs generally offer higher robustness against wafer‐scale fabrication deviations. To mitigate the narrowband splitting ratio of conventional DCs, novel directional couplers have been introduced, incorporating asymmetric sections in the design [23, 24, 25]. For bent DCs, operation still relies on evanescent coupling between adjacent waveguides; however, the curvature induces a controlled phase mismatch between the inner and outer waveguides due to their different radii of curvature. As a result, complete power transfer is suppressed, and a stable ∼50:50 splitting ratio is maintained over a broad spectral range [23]. This represents a significant improvement compared to conventional straight DCs. Finally, broadband ACs are inherently robust both in insertion loss and the splitting ratio. However, their scalability is limited by footprints reaching several hundreds of microns. To address this issue, shortcuts to adiabaticity have been introduced in the form of rapid adiabatic couplers (RACs), which preserve adiabatic performance while significantly reducing device footprint [26]. Beyond these, more exotic 50:50 splitters like subwavelength grating (SWG) splitters have been reported, showing reliability and scalability constraints due to foundry minimum feature sizes and wafer‐scale fabrication tolerances [22].

Figure 1a illustrates the schematic diagrams and design parameters of the representative 50:50 splitter considered in this work. Each device was designed and optimised to identify the trade‐offs among insertion loss, splitting ratio, footprint and wafer‐scale robustness. Optimisation targeted operation in the O‐band, from 1260 to 1360 nm. The design process combined commercial finite difference eigenmode (FDE) and three‐dimensional (3D) finite difference time domain (FDTD) solvers, whereas optimisation relied on established numerical strategies, including surrogate modelling, principal component analysis (PCA), particle swarm optimisation (PSO) and gradient‐descent methods [27, 28].

**FIGURE 1:**
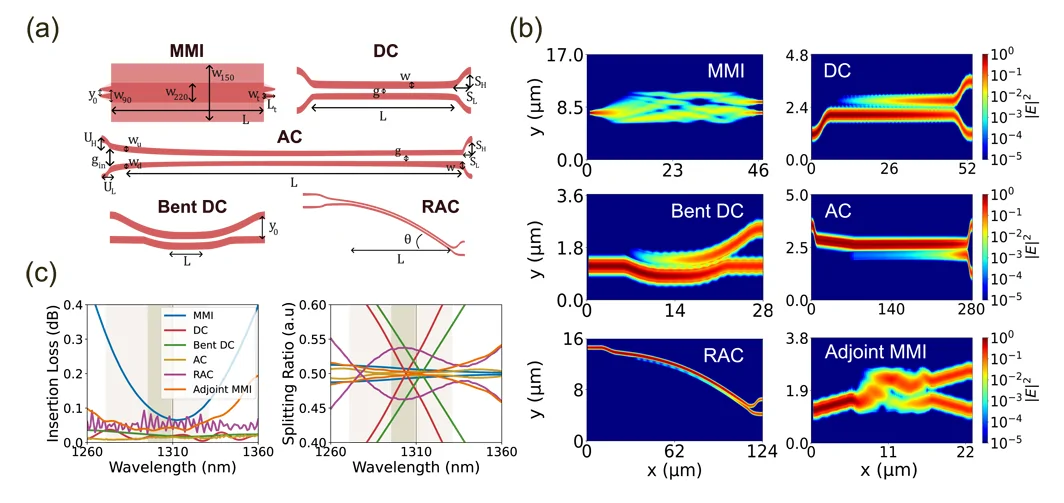
Simulation of 50:50 power splitters. (a) Schematic diagrams and design parameters. Red shades denote silicon layer thicknesses in the 220‐nm SOI platform: 90, 150 and 220‐nm from lighter to darker, respectively. (b) Simulated squared absolute E‐field distributions at mid‐height of the waveguide and 1310 nm for the optimised splitter designs. (c) Simulated insertion loss and splitting ratio spectral responses for the optimised 50:50 splitters. Beige shades from darker to lighter tones emphasise DR4, ER4 and FR4 networking transceiver standards.

#### 2.1.1 Multimode Interferometer

The MMI splitter operates on the basis of the self‐imaging effect, in which the input field is periodically reconstructed through interference between higher order modes. The initial seed was defined by fixing the MMI width $\left(w_{220}\right)$ to the minimum value that allows proper allocation of the access waveguides. The 150‐nm silicon layer width $\left(w_{150}\right)$ is three times larger than the MMI width. The remaining parameters were derived using the analytical relations among the MMI body length (L), MMI access spacing $\left(y_{0}\right)$ and MMI width under paired excitation conditions and an FDE solver [29]. To further minimize insertion loss, 90‐nm cross section transitions were introduced at the access waveguides, smoothing the effective index variation along the propagation axis before injecting light into the interferometric region. The optimisation process resulted in a 45.2 × 15 µ$m^{2}$ MMI with values of $y_{0}$ = 1.76, $w_{t}$ = 1.59, $w_{220}$ = 5.0, $w_{150}$ = 15.5, $w_{90}$ = 3.24, $L_{t}$ = 3.0 and L = 39.2 μm.

#### 2.1.2 Directional Coupler

The DC splitter relies on evanescent coupling between parallel waveguides where power oscillates sinusoidally between them over the coupler length. The initial geometry was defined by selecting a gap (g), consistent with the fabrication constraints and a single‐mode waveguide width (w). Based on these values, the effective indices of the two lowest order super‐modes were calculated using an FDE solver, from which the beating length and the corresponding 3‐dB length were derived [30]. For S‐bends’ access waveguides, an effective radius on the order of several tens of microns was selected to limit bending losses. Later, the coupler length was adjusted to (L) to account for the effective coupling from the access waveguides. The optimisation process resulted in a 52.3×2.9 µ$m^{2}$ DC with values of L = 40.3, w = 0.41, g = 0.24, $S_{L}$ = 6.0 and $S_{H}$ = 0.885 μm.

The bent DC relies on evanescent coupling between adjacent waveguides with a controlled phase mismatch. The design parameters were defined following the methodology in [31] but with two modifications: the access waveguides to the bent coupling region and the inclusion of an additional straight coupling section (L) in the mid‐region connecting the two symmetric bent regions as shown in Figure 1a. For the access waveguides, instead of linking their footprint to the bend angle, we fixed the access waveguide offset $\left(y_{0}\right)$ to be 1.0 μm and derived the corresponding access angle, ensuring that adjacent waveguides are sufficiently separated to avoid coupling. This choice not only minimizes the ΔL between branches but also the overall splitter footprint. For the initial seed, we selected a gap, (g), and waveguide width (w) ensuring single‐mode operation, along with a bending radius (R) and a coupling angle (θ) of the same order of magnitude [23]. During the optimisation process, the primary role of the bending radius was to set the central operation wavelength, whereas that of the coupling angle was to maximise bandwidth. This process resulted in a 27.2×2.1 µ$m^{2}$ bent DC with values of w = 0.41, g = 0.178, R = 54.0, L = 3.5 and $y_{0}$ = 1.0 μm, and θ = 11.7°.

#### 2.1.3 Adiabatic Coupler

The AC operates on the principle of mode evolution, where the optical field remains confined to a single instantaneous eigenmode as the coupler geometry varies gradually, ensuring negligible power transfer to higher order modes. Unlike DCs or MMIs, ACs do not rely on a specific coupling length determined by their cross section analysis; instead, the output power distribution is governed by the adiabatic transition and the waveguide width difference at the output section [32]. For the initial seed, we set the upper $\left(w_{u}\right)$ and lower $\left(w_{d}\right)$ waveguide widths to 0.29 and 0.38 μm, respectively, to guarantee single‐mode operation, together with an input gap $\left(g_{in}\right)$ of 0.60 μm to suppress undesired mode crosstalk between adjacent waveguides. Access S‐shaped waveguide tapers were then introduced to excite super‐modes in a controlled manner. The coupling region (L) was then defined as a linear width transition from the input dimensions to symmetric output widths (w) while keeping the gap (g) constant. At the output, equal waveguide widths and symmetric S‐bends were implemented to achieve a 50:50 splitting ratio. The optimisation process resulted in a 284.2×2.7 µ$m^{2}$ AC with optimal values of $w_{u}$ = 0.29, $w_{d}$ = 0.38, $g_{in}$ = 0.60, $U_{H}$ = 0.70, $U_{L}$ = 9.20, L = 264.0, g = 0.16, w = 0.33, $S_{H}$ = 0.9 and $S_{L}$ = 11.0 μm.

The RAC is an evolution of conventional AC, specifically designed to overcome its main drawback: the large footprint. As in ACs, the structure comprises two single‐mode input waveguide widths, symmetric output widths to achieve a 50:50 splitting ratio, input and output S‐bends, and a constant‐gap coupling region with width transitions. The key principle is the introduction of a geometry‐induced tilt (θ) along the coupling region (L), which suppresses crosstalk to the nearest unwanted mode at each point. This additional degree of freedom enables adiabatic mode evolution to be achieved in much shorter lengths, while preserving the intrinsic robustness and broadband response of standard ACs [26]. The optimisation process resulted in a 124×10.8 µ$m^{2}$ RAC with optimal values of $w_{u}$ = 0.29, $w_{d}$ = 0.38, $g_{in}$ = 0.60, $U_{H}$ = 1.10, $U_{L}$ = 35.0, L = 78.0, g = 0.16, w = 0.33, $S_{H}$ = 0.90 and $S_{L}$ = 11.0 μm and θ = 5.66°.

Figure 1b illustrates the simulated E‐field distribution at 1310 nm and 45°C—as it is the standard temperature for networking transceivers—for all the splitter designed. From these results, low IL and a close 50:50 SR at 1310 nm can be expected as no significant scattered light is observed in the background region, E‐field amplitudes are below $10^{-5}$, and the output ports show comparable intensity levels in the colour scale. IL and SR spectral responses are depicted in Figure 1c from 1260 to 1360 nm for all splitters emphasising DR4, ER4 and FR4 networking transceiver standards—for short, extended and long distance ranges, respectively. DR4 corresponds to a single carrier operating at 1310 nm. ER4 is a narrowband format with four carriers at 1295.56, 1300.05, 1304.58 and 1309.14 nm. Last, FR4 is the most demanding one in terms of bandwidth requirements with four carriers at 1271, 1291, 1311 and 1331 nm. AC and RAC designs show broadband 0.06 dB loss and a SR below 0.537 across these bandwidths, but their footprints limit integration density. DC and bent DC designs show compact footprints and broadband 0.03 dB loss, but their narrowband SR is unacceptable for FR4, being above 0.60. MMI design shows both compact footprint and broadband SR below 0.488 for FR4, being the wavelength‐dependent IL its main limitation. The minimum and maximum peak losses are 0.06 and 0.31 dB in FR4, respectively.

To further improve the MMI's performance we used adjoint shape optimisation, which consists of evolving the initial device geometry by computing the derivative of the figure of merit with respect to the change in the refractive index, over the whole design region [33]. As an initial step for achieving this single 220‐nm silicon layer design, we set its access input width w = 0.41 μm, the MMI body width $w_{220}$ = 1.60 μm, the MMI body length L = 10.3 μm and the access offset $y_{0}$ = 1.41 μm. In this case, total figure of merit (FOM) was defined as a linear combination of the spectral response in FR4 of both output ports for the nominal design and the four corner points associated to the foundry fabrication tolerances. Optimisation converged on a 22×1.9 µ$m^{2}$ adjoint MMI device with smoothly curved edges where both IL and SR are not only optimal for 1310 nm, as shown in Figure 1b, but also for a broadband wavelength range, as shown in Figure 1c. Adjoint MMI shows similar IL values of 0.06 dB and SR below 0.512 for the three networking transceiver standards. To assess the relative sensitivity of the different designs to fabrication variations, a corner analysis was performed using the foundry‐reported 3σ tolerances. The results indicate that the DC is robust in IL but more sensitive in SR, whereas the AC maintains good loss robustness with SR sensitivity comparable to the adjoint MMI. In contrast, the adjoint MMI, despite its compact footprint, exhibits higher sensitivity to IL variations. It should also be noted that simulated IL typically provides a lower bound estimate, because FDTD models usually neglect propagation losses, which are on the order of 1–3.5 dB/cm for narrow waveguides, as well as sidewall roughness scattering. In practice, scattering from imperfect sidewalls can be a dominant loss mechanism, and its impact may vary across device regions and structures. Therefore, while simulations provide useful design guidance, experimental characterisation is required to confirm IL performance.

These simulation results position adjoint MMI as the best 50:50 splitter design strategy towards large‐scale silicon photonic programmable processors as it unblocks high integration density while meeting high performance requirements.

### 2.2 Phase Shifters

The electro‐optic response of the programmable photonic processor is mainly influenced by the phase shifter (PS), which controls the optical phase of light propagating in a waveguide by modifying its effective refractive index. Its key metrics are power consumption $\left(P_{\pi }\right)$, defined as the electrical power required to induce a π‐phase shift, and reconfiguration speed, characterised by the rise and fall times that quantify the intervals for the output power to increase from 10% to 90% and to decrease from 90% to 10% of its maximum value, respectively.

In the literature, the most widespread approaches for integrated PS are electro‐optic PS (EOPS) and thermos‐optic PS (TOPS). EOPSs operate by modifying the refractive index under an applied electric field. They provide operation on the nanosecond scale but are associated with high insertion loss and increased fabrication complexity. These can be achieved through the plasma dispersion effect in doped silicon or the Pockels effect in materials such as thin film ${LiNbO}_{3}$ [34]. In contrast, TOPSs are based on the thermo‐optic effect, in which current‐induced heating of the waveguide modifies the effective index of the guided modes and, consequently, their accumulated phase. These are simpler to implement, introduce negligible loss and show reconfiguration speed on the order of microseconds but require managing the potential thermal crosstalk to adjacent waveguides [35, 36]. Beyond these devices, other mechanisms have been explored, such as phase change materials [37] and piezoelectric phase shifters [38].

Therefore, we focus our study on TOPS, as they represent the most convenient approach for reliable large‐scale silicon photonic programmable processors. Designs consisting of fully etched waveguides are generally preferred for TOPS, as they yield lower $P_{\pi }$ than partially etched ones. In addition, narrow heaters placed directly above the waveguide provide higher efficiency than side heater implementations, while $P_{\pi }$, thermal response, and breakdown current are only weakly dependent on the heater length [39]. Figure 2a illustrates the conventional, vertical‐trenched, and fully suspended TOPS designs. Guided by previous considerations, we use in this paper a 0.12‐μm‐thick TiN heater positioned 2 μm above a 0.41‐μm‐width fully etched waveguide. All heaters are 2 μ‐m wide and 60 μ‐m long, ensuring a suitable balance between performance and footprint. The main distinction among these three designs lies in the introduction of etching steps during fabrication. By surrounding the heated waveguide with air, thermal conductance of the optical waveguide to its surrounding is reduced, enhancing thermal isolation. This improves $P_{\pi }$ and crosstalk performance but slows down the thermal response and lowers the breakdown current, thus highlighting an intrinsic trade‐off between power efficiency and speed. To mitigate this trade‐off, interleaved suspended designs have been proposed, where suspended and nonsuspended regions alternate along the heater length [40].

**FIGURE 2:**
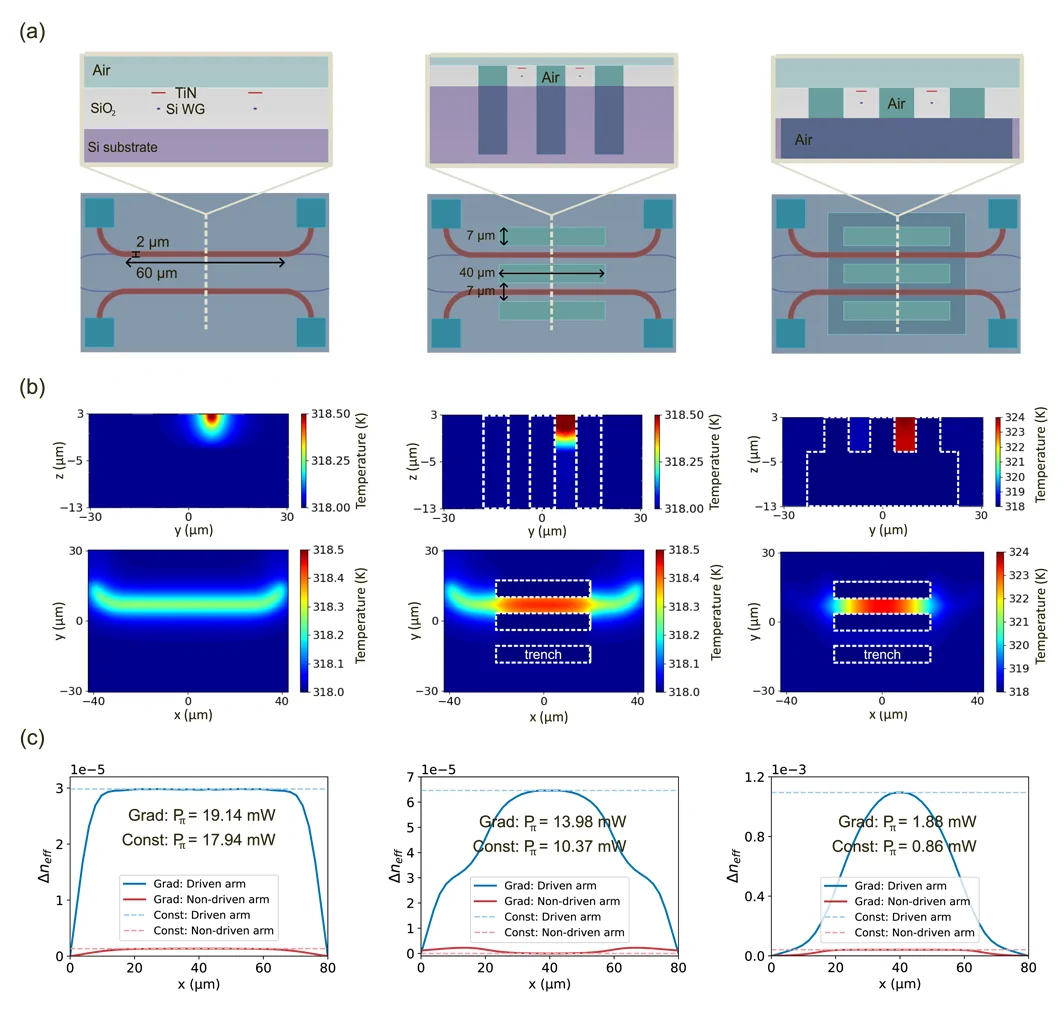
Simulation of thermo‐optic phase shifters. (a) Cross section (upper) and top‐view (lower) schematic of conventional, vertical‐trenched and fully suspended silicon TOPS, from left to right respectively. All three show same TiN heaters, and both trenched designs show same dimensions. (b) Cross section (upper) and top‐view (lower) temperature distribution for the three upper TOPS. (c) Effective refractive index change along the length of both programmable unit cell arms for the three upper TOPS. Power consumption values obtained using gradient and constant models.

Figure 2b shows the simulated temperature distributions for each design, obtained using a commercial three‐dimensional HEAT solver under a 0.25‐V drive voltage and a constant ambient temperature of 45°C (318 K). As all heaters are identical, the same drive voltage implies the same drive electrical power. The cross section distributions confirm that the higher the air confinement around the waveguide, the stronger the thermal isolation, leading to higher temperature rises for the same electrical driven power. In the fully suspended geometry, the maximum temperature rise with respect to the reference temperature reaches 6 K, whereas in the vertical‐trenched geometry, it remains below 0.5 K and in the conventional case, below 0.3 K. Top‐view distributions further illustrate that the thermal gradient along the heater is more pronounced in the fully suspended design, with the maximum temperature located at the heater centre, where thermal isolation is highest. To accurately evaluate $P_{\pi }$, a gradient‐based effective index change model was employed, because it accounts for nonuniform heating and provides a more realistic estimation than simplified uniform‐temperature assumptions, particularly for highly efficient heater geometries.

Multiple thermal cross section monitors were placed at equal intervals along the heater length to discretise the phase shift calculation. For each monitor, the cross‐section temperature distribution was used in an FDE solver to calculate the corresponding local effective index change at 1310 nm. Figure 2c shows the effective index change along the heater for the three cross sections under a 0.25‐V drive. As expected from the temperature maps, the fully suspended geometry exhibits a nonuniform profile, whereas the conventional heater shows a nearly flat trend. The overall phase shift was then obtained by integrating these contributions along the heater length. From this analysis, a $P_{\pi }$ value of 19.14 mW was obtained for the conventional TOPS, 13.98 mW for the vertical‐trenched TOPS and 1.88 mW for the fully suspended TOPS. Conventional heaters exhibit higher $P_{\pi }$ because heat is rapidly dissipated into the substrate. In contrast, suspended heaters suppress thermal leakage by removing both the substrate and surrounding silica, thus enhancing efficiency and significantly reducing $P_{\pi }$. The vertical‐trenched design provides an intermediate case, where silica removal lowers $P_{\pi }$ relative to the conventional design but not to the same extent as the fully suspended geometry. In addition, Figure 2c also illustrates the approximation error introduced when assuming a uniform temperature distribution for each TOPS. This simplification leads to $P_{\pi }$ values of 17.97, 10.37 and 0.88 mW for conventional, vertical‐trenched and fully suspended designs, respectively, which correspond to relative errors of 6%, 26% and 54%, respectively. As expected, the discrepancy is more pronounced for the fully suspended geometry, where the temperature distribution is highly nonuniform.

These results confirm that the fully suspended TOPSs with shared trenches are suitable designs for low power consumption, enabling at the same time a compact 2×2 programmable unit cell with 250×56 µ$m^{2}$ footprint.

### 2.3 Crossings

Besides 2×2 programmable unit cells constituted by 50:50 splitters and phase shifters, crossings are the other key components for large‐scale programmable photonic processors. They provide flexibility when routing optical waveguides in complex photonic chip layouts. Crossings not only limit system optical bandwidth, loss and crosstalk but also scalability. Main performance metrics are insertion loss (IL) and crosstalk (XT). IL accounts for the fraction of optical power transmitted though the desired port, whereas XT establishes how this power is leaked through the undesired ports. A crossing is considered broadband when both the IL and the XT exhibit limited wavelength dependence, thereby ensuring efficient signal transmission and suppressed parasitic coupling over a wide spectral range. Regarding scalability, the main challenges involve both footprint and robustness.

In literature, three main families of silicon crossings can be identified: multimode interferometers (MMIs) based on self‐imaging, beam shaping (BS) based on wavefront deformation and Gaussian‐beam (GB) synthesis based on mode expander theory [41, 42, 43, 44]. All these approaches offer robust performance and a moderate footprint. In terms of losses, MMI‐based crossings exhibit moderate values along with moderate broadband performance. BS techniques offer slightly improved behaviour in both respects. GB synthesis provides the lowest insertion loss combined with broadband operation. Regarding crosstalk, all three strategies show similar performance, typically ranging from −40 to −60 dB. A key differentiating factor when optimising crosstalk is the use of partially etched waveguides, which help suppress diffraction effects. In that case, the intersection region acts as a minor perturbation to the propagating light, so crosstalk can be reduced to below −60 dB [43, 45]. In addition to these families, subwavelength grating (SWG) crossings have been proposed [46, 47], though they tend to be sensitive to fabrication variations. There are also approaches based on Bloch modes in crossing arrays [48, 49], which could become an interesting strategy for scaling once an optimal design is established and validated.

Figure 3a illustrates the schematic diagrams of the representative crossings considered in this work. In the GB PE schematic, two red shades denote the patterned silicon layers of 150 and 220 nm from lighter to darker, respectively. Each device was designed and optimised for FR4 to identify the trade‐offs among insertion loss, crosstalk, footprint and wafer‐scale robustness. The design process combined three‐dimensional (3D) finite difference time domain (FDTD) solvers, while optimisation relied on established numerical strategies, including surrogate modelling, principal component analysis (PCA), particle swarm optimisation (PSO) and gradient‐descent methods [27, 28].

**FIGURE 3:**
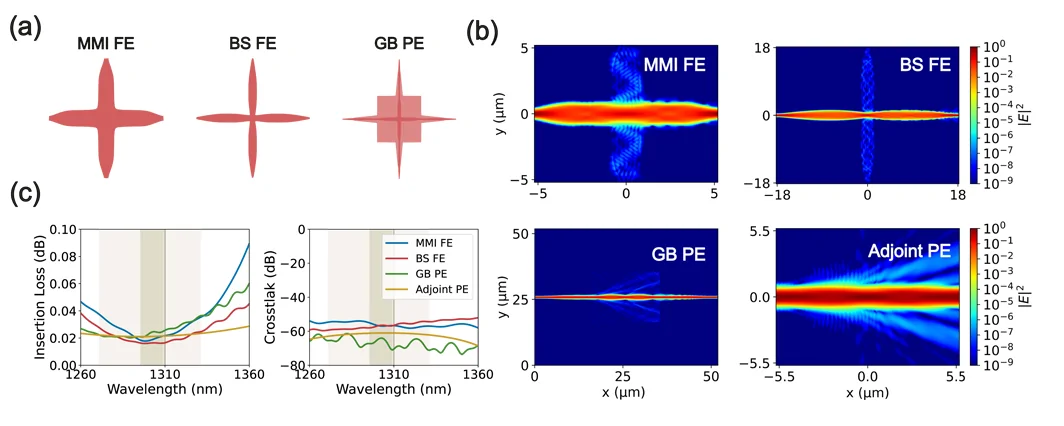
Simulation of silicon waveguide crossings. (a) Schematic diagrams and design parameters. Red shades denote these silicon layer thicknesses in the 220‐nm SOI platform: 150 and 220‐nm from lighter to darker, respectively. (b) Simulated squared absolute E‐field distributions at mid‐height of the waveguide and 1310 nm for optimised crossings. (c) Simulated insertion loss and crosstalk spectral responses for optimised crossings. Beige shades from darker to lighter tones emphasise DR4, ER4 and FR4 networking transceiver standards.

#### 2.3.1 Multimode Interferometer

The MMI‐based crossing consists of a multimode waveguide with a width evolution and is connected on both sides of single‐mode input/output waveguides. Due to the self‐imaging effect, the input field is periodically replicated along the propagation direction. When light is injected from the input waveguide, only even symmetric modes are excited, leading to a symmetric field distribution. As it propagates, the E‐field diverges in the first half of the MMI, refocuses at its centre—corresponding to the intersection with the orthogonal branch—and then diverges again before being efficiently recoupled into the output single mode waveguide. We defined design parameters following the same notation as in [50, 51] and obtained a 11×11 µ$m^{2}$ MMI crossing with optimal values of $w_{1}$ = 0.41, $w_{2}$ = 0.627, $w_{3}$ = 0.627, $w_{4}$ = 0.972, $w_{5}$ = 1.260, $w_{6}$ = 1.440, $w_{7}$ = 1.515, $w_{8}$ = 1.480, $w_{9}$ = 1.518, $w_{10}$ = 1.543, $w_{11}$ = 1.562, $w_{12}$ = 1.766, $w_{13}$ = 2.137, $w_{14}$ = 2.137 and length = 5.5 μm.

#### 2.3.2 Beam Shaping

The BS crossing is implemented using two cosine‐shaped waveguides connected on both sides to single‐mode ones. The curved cosine profile introduces a controlled deformation of the optical wavefront, effectively acting as an integrated lens that redistributes both the field amplitude and the phase [43]. The design parameters were defined following the methodology in [43] but with the addition of a circular region of radius (r) centred at the intersection point. We obtained a 36×36 µ$m^{2}$ crossing with optimal values of $w_{in}$ = 0.41, $w_{\mathrm{out}}$ = 1.49084, $w_{3}$ = 2.70806, $L_{p}$ = 17.1 and r = 0.8$w_{\mathrm{out}}$ µm.

#### 2.3.3 Gaussian Beam Synthesis

The GB synthesis crossing is designed following the mode expander theory, in contrast to conventional MMI‐based crossings. In GB synthesis approach, the input single‐mode waveguide undergoes nonadiabatic transitions that excite multiple higher order modes. Controlled phase shifts are then introduced between these modes, leading to constructive interference that reconstructs a Gaussian‐like beam with a beam waist comparable to the intersection region [44, 52]. The design parameters were defined following the methodology in [52] for the elliptical parabolic taper type. The optimisation process yielded optimal values of $w_{1}$ = 0.70, $w_{2}$ = 1.03, $w_{3}$ = 1.58, $w_{4}$ = 1.55, $L_{1}$ = 1.67, $L_{2}$ = 1.5, $L_{3}$ = 4.75 and $L_{4}$ = 0.7 μm. In addition, 15‐μm cross section transitions were incorporated to this design to ensure that the access waveguides had the same cross section and a 0.41‐μm waveguide width as in the other crossing designs. This results to a 49×49 µ$m^{2}$ Gaussian‐beam crossing exhibiting an additional loss of approximately 0.02 dB per cross section transition.

Figure 3b illustrates the simulated E‐field distribution at 1310 nm and 45°C for all the crossings designed. Low IL at 1310 nm can be expected for all as no significant scattered light is observed in their background region; E‐field amplitudes were below 10^−5^. For FE crossings, light clearly propagates in the undesired vertical direction, whereas for PE crossing, this remains below E‐field amplitudes of 10^−9^. From these results, crosstalk at 1310 nm for FE crossings is expected to be higher than for PE one. IL and XT spectral responses are depicted in Figure 3c from 1260 to 1360 nm for all crossings. Both MMI and BS fully etched crossings exhibit unacceptable crosstalk levels above −60 dB, whereas for GB partially etched crossing, it remains below this limit across the entire spectral range. MMI crossing also exhibits poorest IL spectral response due to its strong wavelength dependence and 0.020 dB IL difference between minimum (0.017 dB) and maximum (0.037 dB) values in FR4. Although beam shaping crossing slightly improves the limitation of 0.01 dB loss difference, the Gaussian beam approach emerges as the most promising among all if extra cross section transition losses are not considered for the comparison. Hence, we propose partially etched waveguide crossings optimised with adjoint shape algorithms [33] as the most promising ones when broadband system performance and large‐scale integration are required.

For the adjoint‐based crossing optimisation, the overall FOM was defined as a linear combination of the spectral responses in FR4 of the transmission and crosstalk ports, considering all the nominal design and the corner cases associated with foundry fabrication tolerances. As an initial design step, the input waveguide width was set to $w_{in}$ = 1.20 μm, the output waveguide width to $w_{\mathrm{out}}$ = 1.20 μm, and the crossing arm length to $L_{1}$ = 4.9 μm. Optimisation converged on an 11×11 µ$m^{2}$ adjoint crossing with smooth edges where both IL and XT are not only optimal for 1310 nm, as shown in Figure 3b, but also for a broadband wavelength range, as shown in Figure 3c. Adjoint PE crossing shows flat IL and XT responses with IL values of 0.023 dB and XT values below −61 dB for the three networking transceiver standards. Fabrication variability of the crossing designs was further evaluated through corner analysis under the foundry‐reported 3σ tolerances. The MMI FE exhibits the highest sensitivity in IL, the BS FE shows intermediate behaviour and the adjoint PE remains the most tolerant among the evaluated designs.

These simulation results position adjoint crossing as the best design strategy towards large‐scale silicon photonic programmable processors as it unblocks high integration density along with meeting high performance requirements.

## 3 Experimental Results

In this section, we define wafer‐level test structures for 50:50 splitters, 2×2 programmable unit cells and crossings’ characterisation. Then, we assess their wafer‐scale performance against networking transceiver standards.

### 3.1 50:50 Splitters

Two 50:50 splitter designs were fabricated: one based on a typical MMI optimised with standard algorithms and another obtained through adjoint shape optimisation. Figure 4a illustrates two structures intended for 50:50 splitters’ characterisation using cutback methodology. In both, to avoid spurious fringes caused by Fabry–Perot cavities, the inter‐splitter distances are randomised considering the effective index of the connecting waveguides, preventing strong periodicities that could mask the true spectral response. Additional control loops help to monitor grating coupler (GC) variability and identify unexpected process deviations. For both configurations, the use of vertical GCs, a tuneable laser, a fibre array, an automated XY table and hexapod measurement procedure enabled robust and scalable wafer‐level testing of 50:50 splitters.

**FIGURE 4:**
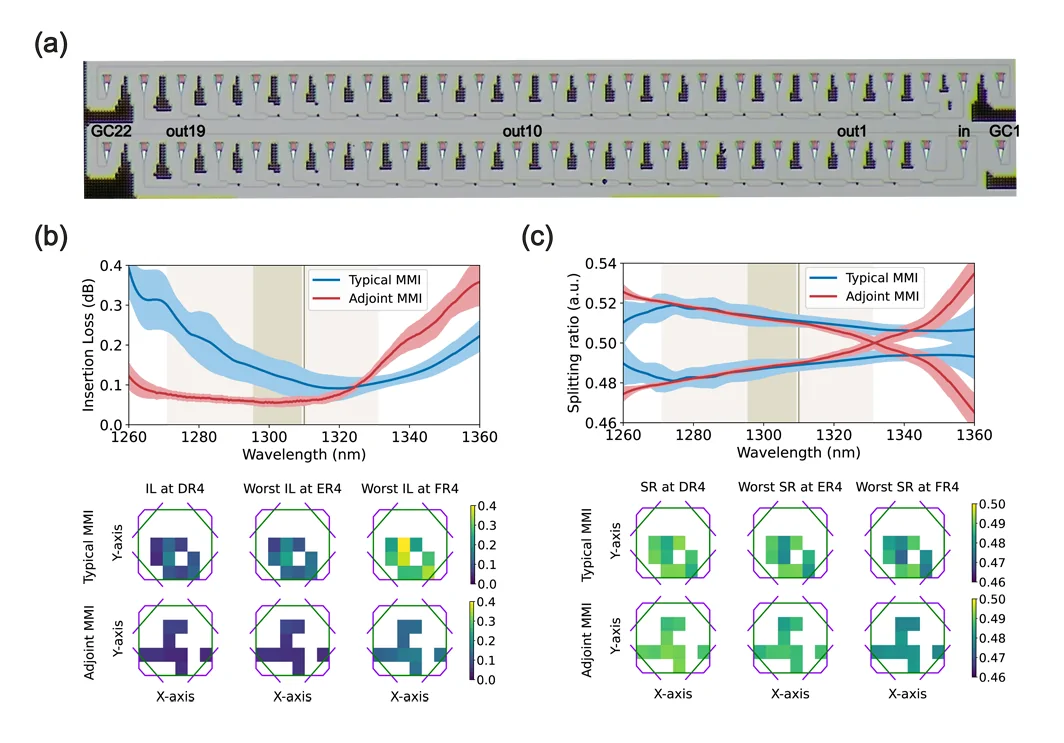
Experimental measurements of 50:50 splitters. (a) Microscope image of the fabricated wafer‐scale test structure for 50:50 splitter characterisation. In the upper structure, 20 cascaded splitters are connected through their bar ports, with the cross port of the *i* + 1th splitter connected to the *i*th output GC. In the lower structure, 20 cascaded splitters are connected through their cross ports and with their bar ports to the output GCs. Additional GCs (GC1–GC22) act as a control and normalisation loops. (b) Upper: Wafer‐scale statistical insertion loss spectral response for typical and adjoint MMI splitters. Mean values are represented as solid lines, and standard deviation as shaded regions. Background beige shades from darker to lighter tones emphasise DR4, ER4 and FR4 networking transceiver standards. Lower: Wafer‐scale spatial distribution of worst insertion loss in DR4, ER4 and FR4 for typical and adjoint MMI splitters. Each square represents a die. (c) Upper: Wafer‐scale statistical splitting ratio spectral response for typical and adjoint MMI splitters. Mean values are represented as solid lines, and standard deviation as shaded regions. Background beige shades from darker to lighter tones emphasise DR4, ER4 and FR4 networking transceiver standards. Lower: Wafer‐scale spatial distribution of worst splitting ratio in DR4, ER4 and FR4 for typical and adjoint MMI splitters. Each square represents a die.

The cutback method estimates the wavelength‐dependent loss of the device under test (DUT) from the slope of a linear regression Y(X), where Y represents the normalised output power at each wavelength and X the number of cascaded devices. To avoid overestimation, normalising to non‐DUT–dependent losses that increase linearly with the number of cutback stages is essential. Neglecting normalisation of the connecting waveguides of the cascaded splitters may introduce errors up to ∼60% in the estimated IL, given propagation losses of ∼3.5 dB/cm. This value was obtained from our experimental characterisation of a single‐mode waveguide, in good agreement with the foundry specifications, and could be reduced to ∼1 dB/cm in alternative foundry platforms or below 1 dB/cm for multimode waveguides. However, no GC loss normalisation is required because the number of GCs is constant between the cutback stages (two per stage input and *i*th output GC) and their variability remains well controlled. In FR4, the standard deviation is below 0.5 dB, supporting the assumption that all GCs are fabricated identically across the cross‐ and bar‐cascaded splitters. Finally, only (X,Y) pairs with output powers above the noise floor of the power monitor (−70 dBm) are considered for the linear regression. This ensures that estimated slope is not corrupted. Following this procedure, splitter total bar $\left(TL_{b}\right)$ and cross $\left(TL_{c}\right)$ losses are obtained from the upper and lower structures shown in Figure 4a, respectively. Those values account for both IL and SR contributions per port, respectively, related as

$$IL=-10\log_{10}\left(10^{TL_{b}/10}+10^{TL_{c}/10}\right)$$

and

$$SR_{b,c}=10^{TL_{b,c}/10}/\left(10^{TL_{b}/10}+10^{TL_{c}/10}\right).$$

Figure 4b shows the wafer‐scale statistical spectral response of the insertion loss (IL) for both designs. In total, nine reticles were measured, as the running mean of the IL—defined as the cumulative average of the first N measured reticles and evaluated over multiple random orderings—stabilises from N∼6–7 and remains within confidence bounds beyond N∼8–9, thereby reducing measurement time without compromising statistical reliability. In the upper panel, narrow shaded regions indicate low variability and robust tolerance, whereas wider regions reflect greater sensitivity to process fluctuations. In the lower panel, wafer maps display worst case DR4, ER4 and FR4 IL spatial distribution. Uniform colour distribution across the wafer suggests good fabrication uniformity or/and device robustness, whereas localised variations point to sensitive devices to process deviations. For both designs, the IL standard deviation is lower near the minimum loss wavelengths and increases towards the spectral edges. This behaviour arises from the parabolic shape of the MMI IL spectrum: The flatter slope at the minimum makes the device less sensitive to deviations, whereas the steeper slopes at the edges increase the impact of fabrication errors. Additional variability at extreme wavelengths is also influenced by the spectral response of the GC used for I/O coupling. Typical MMI shows the worst wafer‐scale mean IL of 0.10 ± 0.04 dB in DR4, 0.14 ± 0.04 in ER4 and 0.30 ± 0.06 dB in FR4, whereas adjoint MMI shows worst wafer‐scale mean insertion loss of 0.06 ± 0.01 dB in DR4, 0.06 ± 0.01 in ER4 and 0.14 ± 0.01 dB in FR4. Thus, adjoint MMI clearly outperforms typical MMI by not only flattening spectral response and lowering losses but also by diminishing its fabrication variability.

Figure 4c presents a similar analysis for the splitting ratio (SR). The splitting ratio of typical MMI shows the worst wafer‐scale mean value of 0.489 ± 0.003 in DR4, 0.486 ± 0.005 in ER4 and 0.482 ± 0.007 dB in FR4, whereas adjoint MMI shows the worst wafer‐scale mean value of 0.490 ± 0.001 in DR4, 0.486 ± 0.001 in ER4 and 0.479 ± 0.001 dB in FR4. Both exhibit similar performance against the transceiver standards, but the adjoint design considerably improves fabrication sensitivity.

These wafer‐scale experimental results demonstrate the robustness and broadband performance of the adjoint shape‐optimised MMI, positioning it as a compact 50:50 splitter for reliable and scalable silicon photonic programmable processors.

### 3.2 2×2 Programmable Unit Cells

Two 2×2 programmable unit cells (PUCs) were fabricated as shown in Figure 5a, one with a 380×150 µ$m^{2}$ footprint using a 200‐μm TOPS and other with a 250×56 µ$m^{2}$ footprint using a 60‐μm TOPS, both above a narrow waveguide. One integrates two independent fully suspended trenches and a 200‐μm titanium nitride (TiN) heater, and the other shared fully suspended trenches and a 60‐μm TiN heater. Electro‐optic characterisation was performed by driving the phase shifter with an arbitrary waveform generator (AWG) through a multi‐contact probe. The injected voltage modulated the optical output power between the two MZI output ports, and rise–fall times were extracted from the temporal response of the signal. Optical characterisation employed single fibres, a broadband source and an optical spectrum analyser (OSA). Output spectra were recorded with the OSA under fixed phase shifter currents, and then, the responses were normalised to a GC monitor loop similar to the one described previously. Transmission and crosstalk were obtained for the bar and cross states, defined as the current conditions maximising transmission from in0 → out0 and in0 → out1, respectively, with crosstalk measured as the residual power at the complementary port. The switching power $P_{\pi }$ was computed as the electrical power difference required to transfer the minimum optical power from one output port to the other. Although it can also be derived from the maxima of the switching response, using the minima yields improved accuracy due to their steeper spectral slope.

**FIGURE 5:**
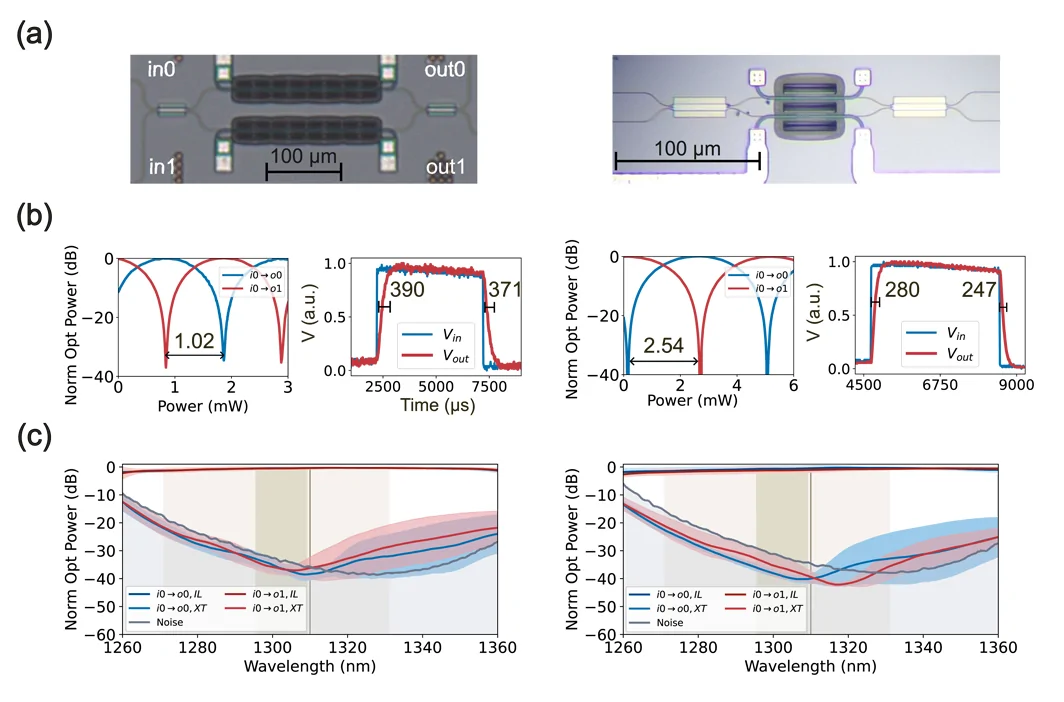
Experimental measurements of 2×2 programmable unit cells. (a) Microscope image of a photonic circuit consisting of two independent (left) 200 μm TOPS or two shared (right) 60 μm TOPS and two typical MMI splitters. (b) Experimental power consumption and rise–fall times at 1310 nm for (left) 200 μm TOPS or (right) 60 μm TOPS PUC. (c) Wafer‐scale statistical insertion loss and crosstalk for bar and cross states for (left) 200 μm TOPS or (right) 60 μm TOPS PUC. Noise corresponds to minimum measurable power above noise floor. Background beige shades from darker to lighter tones emphasise DR4, ER4 and FR4 networking transceiver standards.

Figure 5b shows the $P_{\pi }$ and rise–fall times for these PUCs. The 200‐μm TOPS exhibits a $P_{\pi }$ of 1.02 mW and rise–fall times of 390–371 µs, whereas the 60‐μm TOPS achieves $P_{\pi }$ of 2.54 mW and rise–fall times of 280–247 µs, as shown in middle panels of Figure 5a and b, respectively. Both show comparable efficiency and rise–fall times, whereas the shorter TOPS offers a footprint reduction. In addition, we performed hysteresis tests for the short one showing heater degradation for electrical power values above 12.6 mW. This threshold corresponds to ∼5$P_{\pi }$ cycles, which is enough to calibrate and configure the PUCs.

Figure 5c illustrates wafer‐scale statistical optical responses for IL and XT for both bar and cross states, respectively. Both PUCs show similar optical performance, as it is primarily determined by the 50:50 splitter, which is identically implemented in both cases in a point‐symmetric configuration [53]. They show approximately a similar wafer‐scale worst mean insertion loss of 0.39 ± 0.37 dB in DR4, 0.56 ± 0.54 in ER4 and 1.31 ± 1.67 dB in FR4 for bar and cross states, as expected because the only difference between them is the waveguide routing length. These are consistent with IL spectral response previously reported for the individual components but show higher standard deviations, mainly due to the variability of the GCs and the PUC tuning process. A more reliable estimation can be obtained by accounting for each contributing factor along the routing path: the IL of the two 50:50 splitters and the narrow waveguide propagation loss. Assuming perfect tuning of the PUCs, we obtained worst IL of 0.79 and 0.63 dB for the 200 and 60 μm TOPS PUCs in FR4, respectively. Crosstalk for both PUCs show worst mean values of approximately −36 ± 4 dB in DR4, −34 ± 3 in ER4 and −22 ± 3 dB in FR4 for bar and cross states.

Overall, the 60‐μm TOPS enables four times more compact 250×56 µ$m^{2}$ PUC while preserving power consumption, temporal response and optical performance, confirming its potential for reliable and scalable silicon photonic programmable processors where footprint reduction is critical. On combining previously reported adjoint MMIs with these 60 μm TOPSs, it can be observed that the PUC loss is expected to be 0.13 dB in DR4 and ER4 and 0.30 dB in FR4. Moreover, crosstalk is expected to improve, as the SR remains similar while its standard deviation decreases.

### 3.3 Crossings

Three crossings were fabricated: a typical BS FE, a typical GB PE and an adjoint PE crossing. Figure 6a illustrates two structures intended for crossings’ characterisation one using cutback methodology and the other crosstalk statistical analysis. To suppress parasitic Fabry–Perot fringes, the distances between devices are randomised considering the effective index of the connecting waveguides, thereby avoiding periodic artifacts that could distort the measured spectral response. For both configurations, the use of vertical GCs, a tuneable laser, a fibre array, an automated XY table and hexapod measurement procedure enabled robust and scalable wafer‐level testing of crossings.

**FIGURE 6:**
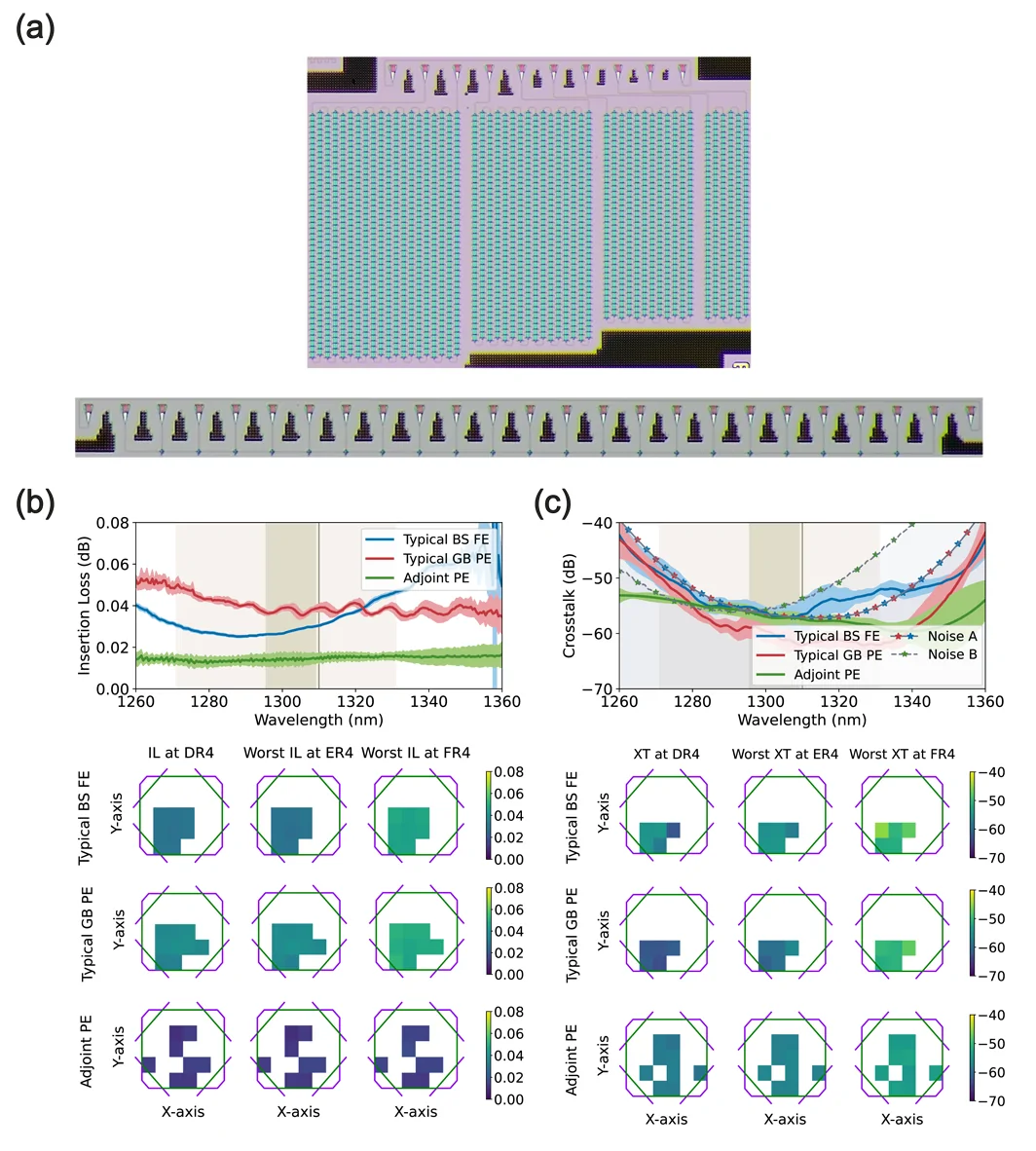
Experimental measurements of crossings. (a) Microscope image of the fabricated wafer‐scale test structure for crossing characterisation. Upper: Test structure for insertion loss characterisation including a GC monitoring loop and four cutback stages consisting of an increasing number of crossings in the order and steps of hundreds. Lower: Test structure for crosstalk characterisation. Twenty crossings are cascaded in transmission, and undesired transmission ports are connected to output GCs using same labelling as in splitter test structure (see Figure 4a). (b) Upper: Wafer‐scale statistical insertion loss spectral response for typical beam shaping fully etched, Gaussian‐beam partially etched and adjoint partially etched crossings. Mean values are represented as solid lines, and standard deviation as shaded regions. Background beige shades from darker to lighter tones emphasise DR4, ER4 and FR4 networking transceiver standards. Lower: Wafer‐scale spatial distribution of worst insertion loss in DR4, ER4 and FR4 for those crossings. (c) Upper: Wafer‐scale statistical crosstalk spectral response for typical beam shaping fully etched, Gaussian‐beam partially etched and adjoint partially etched crossings. Mean values are represented as solid lines, and standard deviation as shaded regions. Noises A and B correspond to minimum measurable power above noise floor. Those are mapped with each crossing set‐up conditions using coloured stars. Background beige shades from darker to lighter tones emphasise DR4, ER4 and FR4 networking transceiver standards. Lower: Wafer‐scale spatial distribution of worst insertion loss in DR4, ER4 and FR4 for typical beam shaping fully etched (BS FE), Gaussian‐beam partially etched (GB PE) and adjoint partially etched (PE) crossings.

Cutback methodology was already described and discussed at 50:50 splitters’ subsections; here, same concerns regarding propagation loss normalisation and noise floor apply. Crosstalk statistics were computed by normalising the spectral response measured at the output GCs to the GC control loop. However, this estimation is limited not only by the GC variability but also ultimately by the sensitivity of the power monitor (−70 dBm), as crosstalk values are expected to approach the same order of magnitude.

Figure 6b shows the wafer‐scale statistical insertion loss spectral response for all designs. In this case, approximately nine reticles were measured for IL extraction and at least five for XT, ensuring efficient measurement with statistical reliability. In the upper panel, narrow shaded regions indicate low variability and robust tolerance, whereas wider regions reflect greater sensitivity to process fluctuations. In the lower panel, wafer maps display worst case DR4, ER4 and FR4 IL spatial distribution for each. Uniform colour distribution across the wafer suggests good fabrication uniformity or/and device robustness, whereas localised variations point to sensitive devices to process deviations. Both typical crossings show wafer‐scale mean insertion loss spectral response wavelength dependent. However, for adjoint crossing, the mean insertion loss spectral response is not only perfectly flat but its values also are up to three times smaller. Typical GB PE crossing shows worst wafer‐scale mean insertion loss with worst mean IL of 0.039 ± 0.002 dB in DR4, 0.039 ± 0.002 in ER4 and 0.049 ± 0.004 dB in FR4. Typical BS FE crossing slightly outperforms it with worst mean IL of 0.0304 ± 0.0005 dB in DR4, 0.0301 ± 0.0004 in ER4 and 0.047 ± 0.001 dB in FR4, whereas adjoint crossing clearly outperforms both with worst mean insertion loss of 0.014 ± 0.002 dB in DR4, 0.015 ± 0.002 in ER4 and 0.016 ± 0.004 dB in FR4. Thus, adjoint PE crossing outperforms typical designs not only by lowering losses but also by flattening spectral response. All three designs show robustness to fabrication because standard deviations are similar and low.

Figure 6c presents a similar analysis for the crosstalk (XT). To compute noise levels, the power monitor sensitivity is normalised to the GC monitoring loop. For typical BS FE crossing, wafer‐scale mean crosstalk could be estimated because it is above the noise floor limit being −56 ± 3 dB for DR4, −55 ± 1 dB for ER4 and ≤ −49 ± 3 dB for FR4. However, in both PE cases, wafer‐scale mean crosstalk is below the noise limit, which corresponds to the lowest power that can be distinguished from the random fluctuation of noise, for the whole spectra. Then, their exact values cannot be resolved, and the only rigorous statement that can be made is that the crosstalk is in each case lower or equal than its noise level, so it sets its upper bound. From that, the typical crossing mean crosstalk values are expected at least to be ≤ −57 ± 1 dB for DR4, ≤ −56 ± 1 dB for ER4 and ≤ −47 ± 1 dB for FR4, whereas the adjoint ones were expected to be ≤ −54 ± 1 dB for DR4, ≤ −54 ± 1 dB for ER4 and ≤ −46 ± 1 for FR4. Therefore, FE crossing shows higher crosstalk values than PE crossings. For PE crossings, wafer‐scale statistical crosstalk values are below the noise level and deviations are mainly due to the GCs variability.

These wafer‐scale experimental results demonstrate the robustness and broadband performance of the partially etched adjoint shape‐optimised crossing and positions it as a suitable and compact silicon crossing option for reliable and scalable silicon photonic programmable processors. For very large‐scale circuits requiring hundreds or thousands of crossings, the use of multilayer silicon nitride‐on‐silicon–integrated photonic platforms may be crucial to unblock loss reduction as these 3D crossings exhibit insertion loss below 2.1 mdB [54, 55].

## 4 Circuit Validation and Discussion

In this section, we experimentally illustrate the circuit‐level impact of the prior components employing a 4‐port Benes optical circuit switch as a processor example. Figure 7a illustrates the fabricated 4×4 photonic switch fabric using a Benes architecture. Its validation was carried out using two single fibres (SFs) with XYZ translation stages, a broadband light source, a multi‐contact probe for driving the PUCs and an optical spectrum analyser. For each spatial permutation, we tuned the PUCs to maximise optical power at the desired output while suppressing crosstalk into undesired ports. Figure 7b shows the optical spectral response of the sixteen input–output spatial permutations presenting a complete 4×4 power transfer matrix in terms of IL and XT. In this matrix representation, the main diagonal corresponds to the identity permutation, whereas the secondary diagonal represents the inverse permutation. The noise floor limit for each trace is determined by the GCs, the OSA sensitivity, and the tilt angle of the SF with respect to the normal of the photonic integrated circuit (PIC) surface. Most of the matrix elements show a unique noise level corresponding to all traces. However, in1–out3 permutation shows two noise levels: Noise A corresponding to i1 → {o3, o4} and Noise B corresponding to i1 → {o1, o2}. This originates from the measurement process, during which one of the fibres was damaged and had to be replaced. The realignment modified the tilt angle of the SF, which in turn shifted the spectral response of the GC.

**FIGURE 7:**
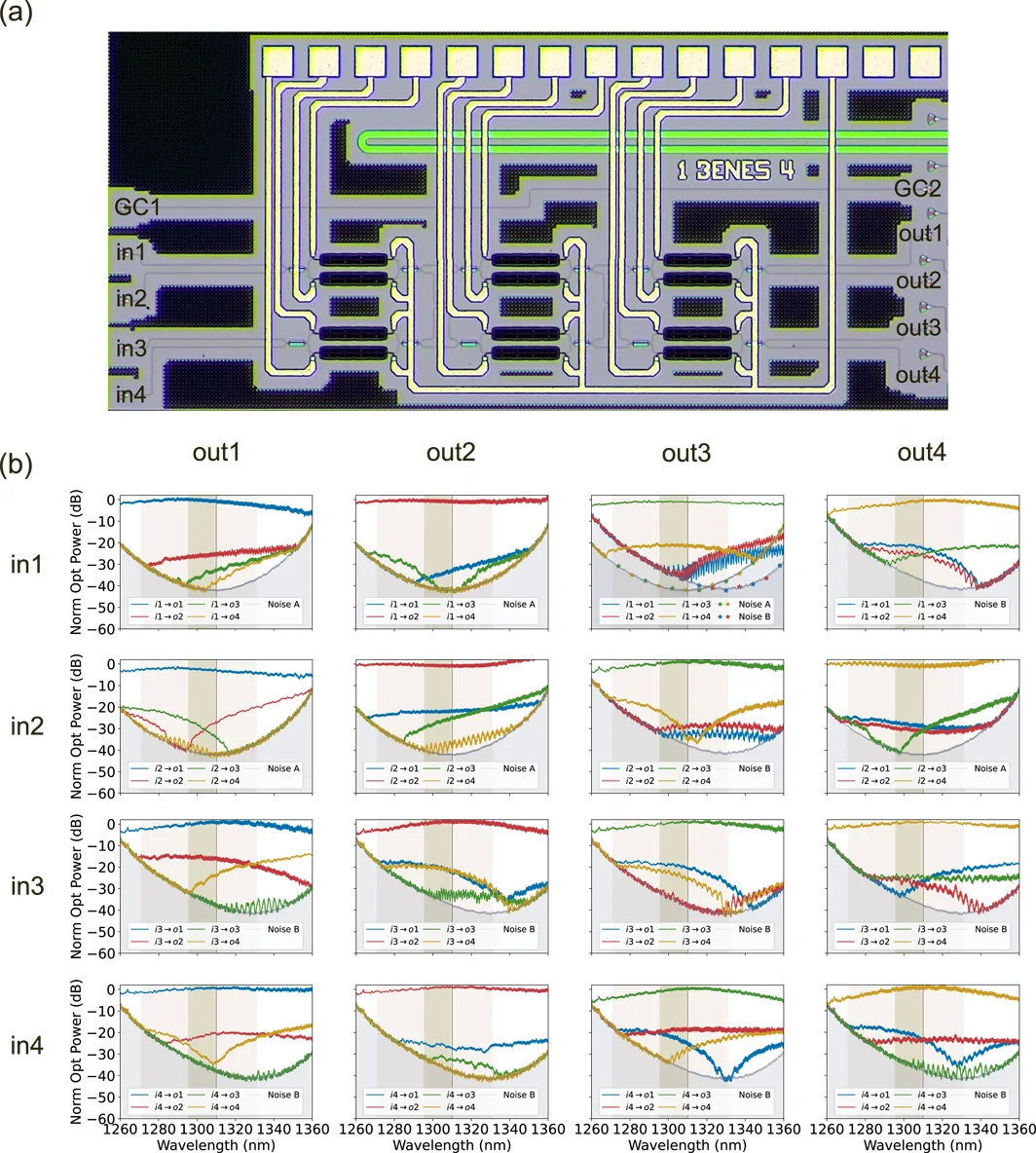
Experimental characterisation of the 4×4 Benes switch circuit. (a) Microscope image of the fabricated photonic circuit. It comprises four input (in1–in4) and four output (out1–out4) GCs, six 2×2 thermo‐optic MZI PUCs, consisting of 200 μm suspended TOPS and typical MMI 50:50 splitters and two typical MMI FE crossings. In addition, there is a normalisation loop connecting GC1 and GC2. The light yellow squares at the top are the electric contacts for driving the PUCs. (b) Experimental spectral response of the sixteen input–output spatial permutations presenting a complete 4×4 power transfer matrix. Each matrix element contains up to six traces for IL, XT and noise representation. IL is given by the trace where input and output match the corresponding matrix indices, whereas the remaining input–output traces correspond to XT from the input signal leaking into the other output ports. The rest of them represent the noise floor limit for each trace. In case there are different levels, they are mapped with each trace set‐up conditions using coloured stars. Background beige shades from darker to lighter tones emphasise DR4, ER4 and FR4 networking transceiver standards.

We estimated that for these sixteen input–output spatial permutation measurements, the worst mean IL was 0.05 ± 1.11 dB in DR4, 0.43 ± 0.78 in ER4 and 1.94 ± 1.32 dB in FR4. These are consistent with IL spectral responses previously reported for the individual components but show higher standard deviations, mainly due to the variability of the GCs and the PUC tuning process. Using IL values of the PUCs and crossings previously reported and computing the narrow waveguide propagation loss, an analytical IL estimation can be performed. Assuming perfect tuning of the PUCs and by accounting for each contributing factor along the connecting path, we obtained worst ILs of 1.57 dB in DR4, 1.87 dB ER4 and 2.85 dB in FR4. In this case, this approach provides a more realistic IL value that is tied to the architecture itself and independent of GC or tuning variability. We also estimated worst mean XT for the 48 undesired leaked powers to be −32 ± 11 dB in DR4, −33 ± 14 dB in ER4 and −33 ± 15 dB in FR4, where coarse PUC tuning is responsible for large standard deviations. These values are consistent with first‐order crosstalk architectures where the PUC crosstalk is the main limitation. Finally, the mean total power consumption for this 4‐port Benes remained below 15 mW across all permutations, consistent with six PUCs of $P_{\pi }\;∼$1.0 mW.

Then, we connected a 400 Gbps LWDM (ER4) transceiver (TRx) and measured BER values for stable data transmission across the sixteen input–output Benes spatial permutations. BER values range from 9.72 $\times 10^{-7}$ to 3.81 $\times 10^{-6}$, with reference value from a back‐to‐back link of 3.21 $\times 10^{-8}$ and from the normalisation loop of 1.27 $\times 10^{-7}$. The observed BER degradation mainly originates from the power imbalance among carriers, which is not caused by the intrinsic performance of the silicon photonic programmable processor (see Figure 7b) but rather by the limited spectral bandwidth of the GCs. The use of edge couplers (ECs) will typically enhance overall system performance, by providing broadband and polarisation‐stable coupling and will also serve as a key enabler for large‐scale photonic packaging. These results confirm the correct routing functionality of the 4‐port Benes optical circuit switch.

Its performance may improve with the design of state‐of‐the‐art, compact and reliable building blocks. To this end, adjoint designs have already demonstrated superior wafer‐scale robustness and performance for high‐integration splitters and crossings, as shown in Figures 4 and 6.

Polarisation management is also a critical component of the design of programmable photonic processors. Several strategies have been investigated in literature to handle both on‐chip polarisations. The most generic and potentially compact solution is the use of polarisation‐insensitive building blocks [56]. However, achieving polarisation insensitivity at the component level remains highly challenging, as it requires precise design and optimisation to ensure equivalent performance for TE and TM modes. A second approach is polarisation diversity, in which the circuit is divided into two parallel duplicated TE systems. A polarisation beam splitter and rotator (PBSR) separates the two polarisations, converts the TM component to TE and routes both as TE‐polarised signals into the parallel systems. After switching, the output signals are recombined using a polarisation beam rotator and combiner (PBRC) [57]. This strategy provides broadband and robust operation but at the expense of increased footprint and component density, as well as stricter requirements on low‐loss, polarisation‐insensitive ECs, PBSR and PBRC. Finally, in specific scenarios such as wavelength division multiplexing (WDM) schemes where carriers share a common polarisation state—or fixed interleaved states—broadband polarisation controllers can be employed. These devices split, rotate and recombine polarisations to maximise TE transmission [58]. In such systems, compensating for modal polarisation delays is essential to preserve signal integrity.

## 5 Conclusions

In conclusion, we have provided a comprehensive review of the main building blocks that constitute a silicon photonic programmable processor. By demonstrating wafer‐scale reliability, high‐integration density and broadband performance for splitters, crossings and programmable unit cells, we set a clear blueprint for future silicon photonic programmable processors capable of meeting the stringent requirements of emerging industrial applications. We have designed and validated a 22×1.9 µ$m^{2}$ 50:50 splitter exhibiting a wafer‐scale worst‐case mean insertion loss of 0.14 ± 0.01 dB and a splitting ratio of 0.479 ± 0.001 across the FR4 band. A 250×56 µ$m^{2}$ compact 2×2 thermo‐optic MZI PUC with power consumption of $P_{\pi }$ = 2.54 mW and rise–fall times of 280–247 µs. Its optical response delivers wafer‐scale bar state worst‐case mean IL and mean XT of 1.48 ± 1.22 dB and −21 ± 3 dB, respectively, in FR4 and cross state worst‐case mean IL and mean XT of 1.88 ± 1.04 dB and −23 ± 1 dB, respectively, in FR4. A low‐loss and crosstalk 11×11 µ$m^{2}$ crossing providing wafer‐scale worst‐case mean IL and XT of 0.016 ± 0.004 dB and ≤ −46 ± 1 dB, respectively, in FR4. In addition, we have validated the programmable processor functionality as a 4‐port Benes silicon photonic switch circuit at the system level, showing input–output spatial permutation mean IL of 1.94 ± 1.32 dB in FR4 and first‐order XT of −33 ± 15 dB in FR4, which is comparable to that of a single PUC. Besides, we have established high‐speed data transmission using a commercial 400Gbps LWDM TRx and achieving BER values ranging from 9.72 $\times 10^{-7}$ to 3.81 $\times 10^{-7}$ for the 16 spatial permutations.

## Author Contributions

**Cristina Gómez‐Hidalgo:** data curation, investigation, writing – original draft, writing – review and editing, software, formal analysis, methodology, visualization, validation. **Juan Fernández‐Vicente:** investigation, software, formal analysis, writing – review and editing. **Natalia Balbastre‐Benavent:** investigation, validation, writing – review and editing. **María Manglano‐Bermejo:** software, investigation, validation, writing – review and editing. **Marios Papadovasilakis:** investigation, software, formal analysis, writing – review and editing. **Ahmed Elsharabasy:** investigation, software, formal analysis, writing – review and editing. **Rocío Baños‐López:** investigation, validation, writing – review and editing. **Jesús Benítez‐González:** investigation, validation, writing – review and editing. **Ana Gutiérrez‐Campo:** investigation, validation, writing – review and editing. **Tsjerk Hoekstra:** supervision, investigation, project administration, writing – review and editing. **Daniel Pérez‐López:** supervision, funding acquisition, conceptualization, investigation, resources, project administration, methodology, writing – review and editing. **Luis Torrijos‐Morán:** writing – review and editing, investigation, conceptualization, project administration, supervision, resources, visualization, writing – original draft.

## Funding

This work has received funding from the ERC Starting Grant programme under Grant Agreement No. 101076175 (LS‐Photonics Project) and from the HORIZON‐EIC‐2024‐ACCELERATOR under Grant Agreement No. 101217820 (EXCITE Project).

## Conflicts of Interest

The authors declare no competing nonfinancial interests but the following competing financial interests: D.P.L. owns shares at iPronics Programmable Photonics S.L.

## Data Availability

The data that support the findings of this study are available from the corresponding author upon reasonable request.
